# Exploring the Versatility of Azine Derivatives: A Comprehensive Review on Synthesis and Biological Applications

**DOI:** 10.2174/0113895575363243241129100845

**Published:** 2025-01-10

**Authors:** Saeed M.N. Alasmari, Aftab Alam, Fayaz Ur Rahman, Ahmed A. Elhenawy, Abid Ali, Manzoor Ahmad, Momin Khan

**Affiliations:** 1 Department of Biology, Faculty of Science & Arts, Najran University, Najran, 1988, Saudi Arabia;; 2 Department of Chemistry, University of Malakand, Chakdara, Lower Dir, 18800, Pakistan;; 3 Department of Pharmacy, Abbottabad University of Science and Technology, Abbottabad, Khyber Pakhtunkhwa, 22500, Pakistan;; 4 Chemistry Department, Faculty of Science, Al-Azhar University, Nasr City, 11884, Cairo, Egypt;; 5 Department of Zoology, Abdul Wali Khan University, Mardan, 23200, Pakistan;; 6 Department of Chemistry, Abdul Wali Khan University, Mardan, 23200, Pakistan

**Keywords:** Synthesis, di-imines, azines, *bis*-Schiff bases, biological activities, enzyme inhibitions

## Abstract

Organic compounds containing azines, di-imines, or *bis*-Schiff-bases have two azomethine (-CH=N-) functional groups associated with a bridging component. These constituents have attracted attention from a diversity of disciplines, comprising coordination, medicinal, agriculture chemistry, and organic synthesis, because of their comprehensive chemical reactivity and nature. This study determines common synthetic approaches and various biological and pharmacological activities of several substituted *bis*-Schiff byproducts. The usefulness of *bis*-Schiff bases in synthetic chemistry and their potential as inhibitors of a number of enzymes have attracted research attention. We have examined different biological activities and common synthetic methods used to make *bis*-Schiff bases that have been published in the literature. A systematic search of the literature has been performed, and studies fitting the prearranged inclusion standards have been inspected. This review can open up new potentials for upcoming research in this area and advance our information on *bis*-Schiff bases.

## 1 INTRODUCTION

In medicinal/pharmaceutical chemistry, synthetic molecules are crucial since they form the basis for the formation of new medicines and healing agents [1]. To interrelate with specific biological targets, modify biochemical paths, and have the planned pharmacological properties, they are thoroughly synthesized and developed [2]. These substances are necessary for the development and discovery of emerging new drugs as medicinal chemists use them to make molecules that target the biological pathways or disease mechanisms [3]. Researchers have built libraries of numerous compounds by rational synthesis and tested them for varied biological activities to discover lead compounds with therapeutic potential [4]. To develop their selectivity, potency, and pharmacokinetic potentials, these leads have undergone further optimization procedures, including medicinal chemistry techniques, computer modeling, and Structure-activity Relationship (SAR) studies [5]. Due to the expansion of structurally varied molecules with an extensive assortment of stereochemical configurations, chemical scaffolds, and functional groups, synthetic chemistry makes it easier to discover new chemical spaces and find advanced therapeutic applicants [6]. Medicinal chemists can plan molecules with high target affinity and selectivity, reducing off-target effects and enhancing therapeutic outcomes by customizing compounds to interrelate, especially with specific biological properties [7]. In addition to addressing the issue of medication development, synthetic molecules aid in the examination of drug repurposing methods. Lead compounds are formulated into clinical applicants with suitable drug-like characteristics by iterative synthesis and preclinical assessment, easing translation to clinical trials and ultimate usage in patient care [8]. To sum up, synthetic molecules are crucial instruments in medicinal chemistry that push efforts towards optimization, drug discovery, and expansion with the goal of enhancing patient consequences and satisfying frustrated medical supplies [9, 10].

Schiff bases are imines comprising compounds obtained from the condensation of ketones or aldehydes with primary amines. This process was first initiated in 1864 by a German chemist Hugo Schiff around 150 years ago [11]. The common formula of Schiff base is RN=CR_1_R_2_, whereas R, R_1_, and R_2_ might be heteroaryl, alkyl, cycloalkyl, or simple aryl groups, among other likely chemical compounds. The existence of imine bond (-CH=N-) in Schiff bases plays a main role in conversing a comprehensive range of biological abilities to the compounds [12]. The electron-lacking carbon atom and electron-rich nitrogen in imine bond -CH=N- offer powerful binding probabilities with numerous electrophiles and nucleophiles, thus inhibiting targeted diseases or DNA replication [13]. The hydrazone-Schiff base derivatives possess many biological activities, such as anti-convulsant [14], anti-oxidant [15], anti-fungal [16], anti-viral [17], anti-cancer [18], anti-inflammatory [19], analgesic [20], anti-bacterial [21], α-glucosidase [22], urease [23], α-amylase [24], and tyrosinase inhibitors [25].

A group of chemical compounds recognized as *bis*-Schiff bases is well-known to involve the incidence of two Schiff base functional groups (-CH=N-) associated with a spacer moiety [26]. A primary amine and an aldehyde or ketone undergo a condensation reaction to make Schiff bases, which are renowned for their comprehensive variety of structural diversity and miscellaneous chemical reactivity [27]. This condensation reaction occurs twice in the case of *bis*-Schiff bases, leading to two Schiff base influences inside the same molecule. Typically, a diamine chemical reacts with two aldehydes or ketone molecules to produce *bis*-Schiff bases. It is likely to comprise more than a few functional groups and structural motifs in the final molecule by this reaction, which can ensue in trivial conditions and permits elasticity in the choice of starting resources [28]. *Bis*-Schiff bases show a wide-ranging spectrum of chemical characteristics and biological activities, which makes them attractive options for use in a number of disciplines, such as agriculture, organic synthesis, catalysis, material science, coordination, and pharmaceutical chemistry [29]. Due to their probable pharmacological potentials, such as antioxidant, anti-inflammatory, anti-cancer, anti-viral, anti-fungal, anti-bacterial, and enzyme inhibition properties, *bis*-Schiff bases have gained a lot of attention in medicinal chemistry [30]. These molecules, which are attributed to the joint action of numerous Schiff base moieties inside a single molecule, often show augmented biological activity as associated with their monomeric complements. Moreover, structural changes to *bis*-Schiff bases modular design enable fine-tuning their features and exploiting their therapeutic effectiveness [31]. All things measured, *bis*-Schiff bases are a class of organic substances with an extensive range of biological and chemical reactivity. The search for new compounds with possible uses in material science, drug discovery, and other interdisciplinary areas has fueled ongoing research into their synthesis and characterization [32].

This review paper's objective was to present a thorough examination of *bis*-Schiff bases and their uses in medicinal chemistry, with an emphasis on their biological activities. By a thorough review of the literature, we aimed to categorize bis-Schiff bases according to their spacer groups and structural motifs, thereby clarifying the salient characteristics that control their biological effects.

## 2 SYNTHETIC APPROACHES TOWARDS *BIS*-SCHIFF BASES

### 2.1 Synthesis of *Bis*-Schiff Bases in Neutral Condition

**Scheme 1:**
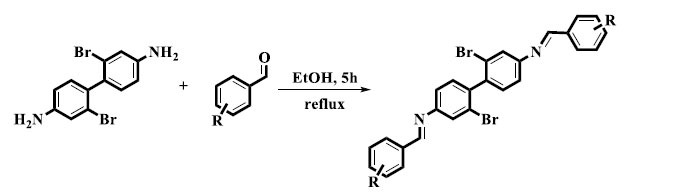
Synthesis of *bis*-Schiff base derivatives in normal conditions.

Without the use of any acid, base, or catalyst, Irfan *et al.* reported the synthesis of *bis*-Schiff base derivatives based on the 2,2'-dibromo-(1,1'-biphenyl)-4,4'-diamine. For five hours, the diamine and several aldehydes were subjected to a reflux reaction while being constantly stirred. There were high yields of the finished products, ranging from 88% to 93% (Scheme **1**). With no additional reagents or catalysts required, this method showed how to synthesize *bis*-Schiff base derivatives quickly and effectively, with potential applications in organic synthesis [33].

### 2.2 Synthesis of *Bis*-Schiff Base Derivatives in Acidic Conditions

**Scheme 2:**
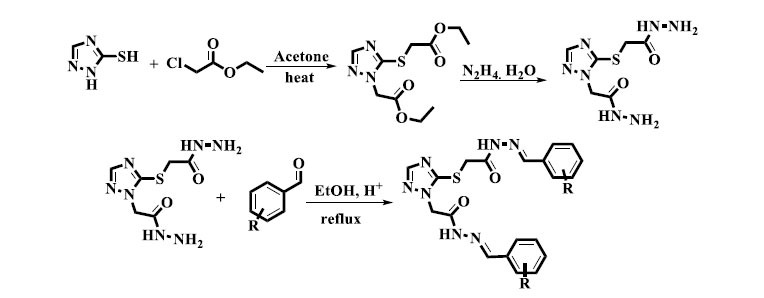
Synthesis of *bis*-Schiff base compounds using acetic acid as a catalyst.

With yields ranging from 85% to 90%, Bushra and her colleagues reported synthesizing new *bis*-Schiff base derivatives with a triazole moiety under acidic reflux conditions. This process probably involved the formation of Schiff bases *via* the condensation of aldehydes or ketones with different amines or hydrazines in the presence of an acid catalyst (Scheme **2**). The *bis*-Schiff base derivatives were then produced by a subsequent condensation reaction with a compound containing triazole. These molecules could be effectively and simply manufactured by means of acidic and reflux conditions, having possible uses in chemistry and medicinal investigation [34].

### 2.3 Ultrasonication Method for the Synthesis of *Bis*-Schiff Base Derivatives

**Scheme 3:**
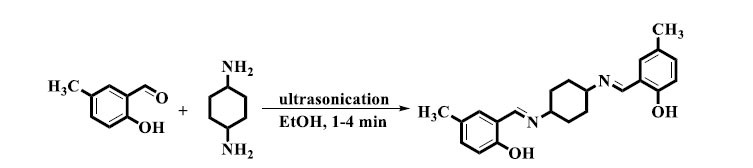
Synthesis of *bis*-Schiff bases through ultrasonication method.

Arafa *et al.* described a novel use of environmentally benevolent sonochemical waves in the production of derivatives of *bis*-Schiff bases. Salicylaldehyde derivatives and diamines were dissolved in 100% ethanol, and then the mixture was immersed in an ultrasonic bath filled with room temperature (25°C) water (Scheme **3**). Afterward, the reaction mixture was unprotected from ultrasound radiation for one to four minutes, and TLC analysis was used to determine whether the reaction was complete. The planned goods were produced quickly and with huge yields. Additional recrystallization in ethanol increased the product yields even further. By providing a quick and easy way to produce *bis*-Schiff base derivatives, this method showed how sonochemistry may be used as an environmentally friendly synthetic process in organic chemistry [35].

### 2.4 Microwave-assisted Synthesis of *Bis*-Schiff Base Derivatives

**Scheme 4:**
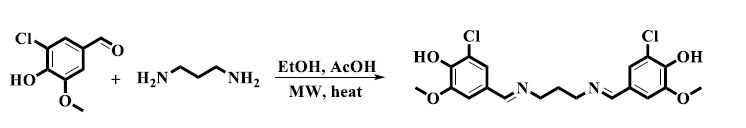
Synthesis of *bis*-Schiff bases *via* microwave-assisted method.

Avinash and his team described employing a microwave-assisted method to produce *bis*-Schiff base compounds. Propane-1,3-diamine, substituted benzaldehyde, and a small amount of acetic acid in ethanol were combined in the synthesis (Scheme **4**). Afterward, this mixture was placed in a microwave reaction vessel with a magnetic stirrer, and the vessel was closed. For the entire three minutes, the reaction was exposed to irregular microwave radiation (power of 50 W) at intermissions of 30 seconds. To obtain the *bis*-Schiff bases, the subsequent solid product was washed with water and then crystallized from 95% ethanol. This microwave-assisted method displayed how microwave irradiation can be used as an ecologically friendly synthetic tool in organic chemistry by providing a rapid and effective way to prepare *bis*-Schiff base compounds (Table **1**) [36].

## 3 BIOLOGICAL ACTIVITIES OF VARIOUS SUBSTITUTED *BIS*-SCHIFF BASES

### 3.1 Anti-glycating Activity

The capability of constituents or interventions to stop the production of Advanced Glycation End products (AGEs), which are related to numeral diseases and the aging process, is known as anti-glycation activity [37, 38]. This method involves discontinuing the non-enzymatic procedure that causes AGE buildup to occur between proteins, sugars, nucleic acids, or lipids. Natural materials, pharmaceutical medicines, dietary antioxidants, and lifestyle alterations are among the chemicals that have anti-glycation activity [39]. These medicines have the potential to reduce the clinical effects of AGE accumulation along with, but not limited to, diabetes problems, cardiovascular illnesses, neurodegenerative complaints, and aging-related disorders, by decreasing the production of AGEs [40]. Harnessing anti-glycation action is a promising therapeutic plan for treating AGE-related illnesses and promoting general health.

In order to examine the possibility of using isatin-based *bis*-Schiff base derivatives as anti-glycating drugs, Khan *et al.* carried out a detailed investigation of their synthesis. The obtained compound **(1)** was found to be an excellent inhibitor of glycation developments for *in vitro* screening. This detection has highlighted the reputation of *bis*-Schiff bases formed from isatin in the formation of inventive medicinal agents that target diseases linked with glycation [41]. Nevertheless, Khan and colleagues described the synthesis and anti-glycation properties of a new benzophenone *bis*-Schiff base derivative **(2)** with hopeful results. The results proved the flexibility of *bis*-Schiff bases made from benzophenone in regulating glycation procedures and suggested their likely use in therapeutic interferences expected in AGE-related ailments [42].


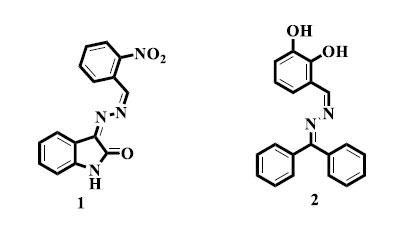


### 3.2 Anti-cancer Activity

Cancer is a multi-faceted disease noticeable by unimpeded cell development and proliferation, which often results in the growth of cancerous tumors [43, 44]. It is associated with a substantial quantity of illness and mortality internationally and can result from a diversity of environmental, genetic, and lifestyle variables [45, 46]. Because cancer has such a terrible effect on both individuals and society as a whole, the need for cancer inhibitors is critical. Anticancer drugs, sometimes referred to as cancer inhibitors, are vital for stopping or delaying the formation of cancer by focusing on particular biochemical pathways that contribute to tumor development and metastasis [47, 48]. These inhibitors may function by preventing the growth of new blood arteries to supply tumors, causing apoptosis, reducing the growth of cancer cells, or interfering with signaling pathways that are essential for the survival and spread of cancer cells [49]. The discovery and development of potent cancer inhibitors are essential to raising patient satisfaction, lowering death rates, and improving patient outcomes.

Ayaz and colleagues designed new derivatives of the *bis*-Schiff base (**3**) and assessed their anti-tumor effects. The most promising activity against malignant glioma was shown by compound *N*'-4-[2-{2-(2,4-dihydroxybenzylidene) hydrazineyl}-2-oxoethoxy]benzylidene-2-(4-isobutylphenyl)pro-paneydrazide (**3**) among the series. This discovery has highlighted the potential of *bis*-Schiff base derivatives as potent cancer therapeutic agents, especially when it comes to aggressive cancer types, like malignant gliomas [50]. Similarly, Jarrahpour *et al.* synthesized an isatin *bis*-Schiff base compound (**4**) and examined it for anti-tumor properties. One of the substances that were examined showed the most potent cytotoxicity against human erythro-leukemia cells. This work has emphasized the potential of isatin-derived *bis*-Schiff base (**5**) as an effective candidate for anti-cancer drugs, with a focus on leukemia [51]. Furthermore, Rani *et al.* reported synthesizing derivatives of *bis*-Schiff bases with promising anti-cancer activity. Their study has further supported the promise of *bis*-Schiff bases as potent anti-cancer agents. This study has added to the increasing amount of data showing the potential of *bis*-Schiff base compounds as promising candidates for the development of anti-cancer drugs [52].


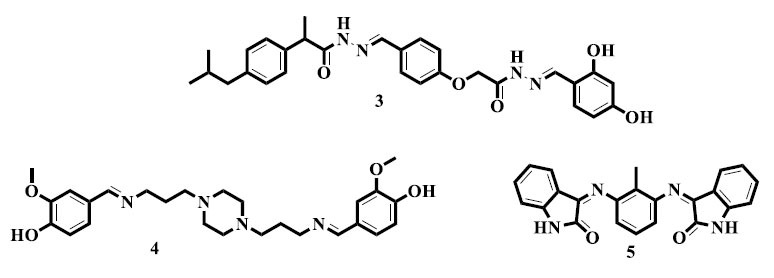


Azine compounds have been examined for their possible anti-cancer mechanisms. One of the published mechanisms of action has been reported to comprise programmed cell death (induction of apoptosis) in cancerous cells [53]. For example, a number of azines underwent interaction with mitochondrial pathways, leading to the activation of caspases and freedom of cytochrome C, being beneficial for the apoptosis cascade [54]. These outcomes involving the inhibition of anti-apoptotic factors and activation of pro-apoptotic factors have endorsed the death of cancerous cells [55]. Moreover, certain studies have recommended that azines may constrain cancer cell proliferation by interfering with the progression of the cell cycle, mainly by blocking the transition to the S phase from the G1 phase [56]. This act can be accredited to the conjugation of key controlling proteins implicated in the cell cycle, like Cyclin-dependent Kinases (CDK) [57].

In addition, azine compounds have been described to display anti-angiogenic properties that can inhibit the growth of tumors by the inhibition of forming new blood vessels by supplying nutrients to the tumors [58]. This result is frequently attributed to the downregulation of Vascular Endothelial Growth Factor (VEGF) and many angiogenic factors [59]. These mechanisms highlight the beneficial potential of azine derivatives in the management of numerous cancers together with destructive ones, like leukemia and glioma (Table **2**) [60-64].

### 3.3 Anti-diabetic Activity

Diabetes, formally referred to as diabetes mellitus, is a long-term metabolic disease marked by high blood glucose levels [65, 66]. This condition is brought on by either inadequate insulin synthesis or poor insulin utilization by the body. The related consequences of this illness have led to epidemic proportions worldwide, with noteworthy implications for public health [67, 68]. Diabetes mellitus raises the chance of a number of grave health issues, such as retinopathy, kidney failure, neuropathy, and cardiovascular disease. Effective therapeutic strategies are desperately needed to manage diabetes and its complications, given the disease's high prevalence and harmful effects [69, 70].

Ahmad *et al.* synthesized *bis*-Schiff base derivatives based on the 4-nitroacetophenone moiety and evaluated their α-glucosidase inhibitory activity. Among the synthetic compounds, 4-[1-(4-nitrophenyl)ethylidene]hydrazonomethyl-benzene-1,3-diol (**6**) exhibited potent inhibitory action [71]. In contrast, promising findings were reported by Ahad and colleagues with regards to the synthesis and α-glucosidase inhibitory activity of substituted benzophenone *bis*-Schiff base compound (**7**) [72].


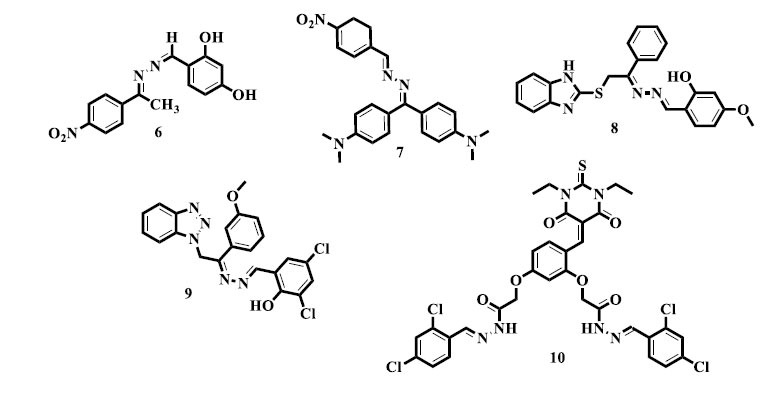


Comparable to this, Rahim *et al.* synthesized special *bis*-Schiff base derivatives with benzimidazole (**8**) nuclei and successfully screened them for α-glucosidase inhibitory activity [73]. Rehman *et al.* also developed a new *bis*-Schiff base product (**9**) with benzo-triazoles as likely anti-diabetic agents. The addition of benzo-triazole moieties proved how flexible this class of substances is for adapting to diabetic ways. The study has highlighted it as vital to examine numerous chemical scaffolds in order to prepare potent anti-diabetic drugs [74]. Eventually, as probable candidates for treating diabetes, Gul *et al.* reported novel *bis*-Schiff base derivatives containing thiobarbituric acid moiety. The study of *bis*-Schiff base compounds (**10**) in the realm of diabetic actions has helped importantly in this effort. The existence of the thiobarbituric acid moiety has drawn attention to the diversity of chemical structures that are being researched for the possibility of stopping diabetes.

### 3.4 Anti-microbial Activity

The capability of substances, comprising pharmaceuticals or natural substances, to defeat or kill microbes, including parasites, fungi, viruses, and bacteria, is known as antimicrobial activity [75]. The deterrence and action of infectious diseases carried on by these pathogens depend heavily on this activity. Antimicrobial drugs can function in a number of different ways, such as rupturing the integrity of the cell membrane, blocking vital metabolic activities, interfering with the synthesis of nucleic acids, or impairing the production of proteins [76, 77]. The creation of potent antimicrobial drugs is crucial for public health, given the mounting threat of antibiotic resistance and the ongoing emergence of novel infectious illnesses. Efforts to battle infectious diseases and protect world health are ongoing and include research on new antimicrobial compounds and the optimization of currently available drugs [78]. Promising results were obtained by Zhu *et al.*, who reported the synthesis and antiviral activity of numerous *bis*-Schiff base (**11**) derivatives and their complexes [79]. Additionally, Abbas *et al.* achieved good results in reporting the synthesis and anti-fungal activity of *bis*-Schiff base derivatives carrying triazole-based lariat macromolecules (**12**). The addition of lariat macromolecules based on triazoles emphasizes the variety of chemical structures being researched for their potential antifungal effects [80]. As possible antibacterial drugs, Ismail and his associates reported synthesizing novel azine compounds based on polyhydroquinolines (**13**). The fact that polyhydroquinolines are used as a scaffold highlights how versatile this class of chemicals is when it comes to drug research [81]. Tantaru *et al.* evaluated the antibacterial activity of *bis*-Schiff bases (**14**) against a variety of bacterial species, producing notable outcomes [82].


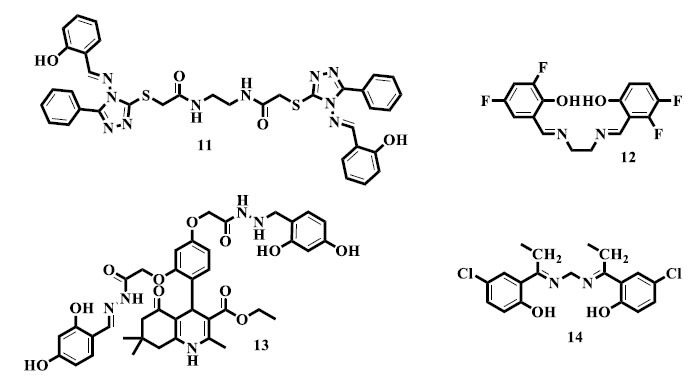


### 3.5 Anti-oxidant Activity

The term antioxidant describes a substance's capacity to counteract or prevent oxidative stress and free radical damage in biological systems [83]. Reactive Oxygen Species (ROS) and Reactive Nitrogen Species (RNS) are examples of free radicals that are produced spontaneously during metabolic processes and in reaction to environmental stressors, such as pollution, UV radiation, and poisons [84]. Free radicals can accelerate aging and cause oxidative damage to lipids, proteins, DNA, and cells when they are present in excess. This can result in a number of disorders [85]. By providing electrons to stabilize free radicals and preventing or lessening their damaging effects, antioxidants combat oxidative stress [86]. They possess the ability to neutralize free radicals, halt chain reactions, and restore biomolecules damaged by oxidation. Numerous natural foods, such as fruits, vegetables, nuts, seeds, and spices, as well as artificial substances, including flavonoids, polyphenols, carotenoids, and vitamins C and E, include antioxidants [87, 88].

As powerful antioxidants, Rashid *et al.* reported synthesizing new *bis*-Schiff base (**15**) compounds with the ethyl phenyl ketone nucleus. Due to the inclusion of ethyl phenyl ketone nucleus, a variety of chemical structures are being researched for their antioxidant potential. The consequences point to these derivatives' potential as influential antioxidants that may have values for stopping diseases associated with oxidative stress and advancing general health [89]. Similarly, Khan *et al.* described the synthesis and antioxidant activity of *bis*-Schiff base (**16**) derivatives bearing isatin nucleus with outstanding results. The potential of isatin in the synthesis of antioxidants is tinted by its usage as a scaffold [90]. Furthermore, interesting results were stated by Demir *et al.* with regards to the synthesis of new *bis*-Schiff base (**17**) derivatives with antioxidant potential [91].


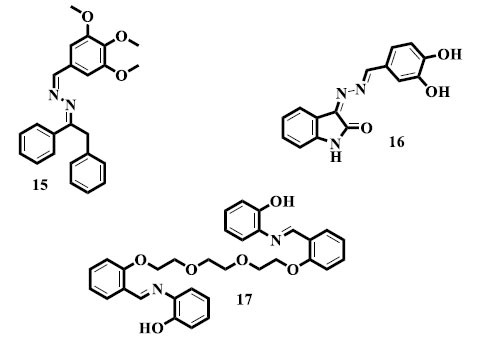


### 3.6 Anti-inflammatory and Analgesic Activities

Pain is a complex and sensual sensation that is often connected to injury or tissue damage [92]. It acts as a safeguard to inform the body of likely danger and set off the proper reactions for self-defense. Acute pain and chronic pain are the two primary categories of pain [93, 94]. Analgesia and inflammation are closely related bodily reactions to injury or infection. The body uses inflammation as a natural defense against stimuli that could cause injury, such as infections, damaged cells, or irritants [95]. Redness, swelling, heat, pain, and even loss of function in the affected area are its hallmarks; when the immune system reacts to a perceived threat by releasing substances, such as prostaglandins and cytokines, inflammation results [96]. Acute inflammation is usually a helpful and protective reaction that aids in the body's healing, but persistent inflammation can cause tissue damage and play a role in the onset of several illnesses, including cancer, heart disease, and arthritis [97]. On the other side, analgesia describes the absence or lessening of pain. Analgesic drugs function by either preventing the nervous system from receiving pain signals or by preventing the inflammatory mediators that cause pain receptors to become more sensitive [98]. Aspirin and ibuprofen are two examples of Non-steroidal Anti-inflammatory Drugs (NSAIDs), which are widely used analgesics with anti-inflammatory qualities. Inhibiting Cyclooxygenase-1 (COX-1) and Cyclooxygenase-2 (COX-2) enzymes lowers prostaglandin synthesis, which is a mediator of inflammation involved in pain signaling [99].

Alam and colleagues conducted a study wherein they synthesized novel *bis*-Schiff base (**18**) derivatives of the commercially available medicine flurbiprofen. The derivatives were then assessed for their *in vivo* anti-inflammatory and analgesic effects, with remarkable outcomes. Flurbiprofen's adaptability in the synthesis of new compounds with improved pharmacological characteristics has been highlighted by its use as a scaffold [100]. Moreover, Sondhi *et al.* produced compounds with double azomethine groups (**19**) and successfully tested them for their ability to reduce inflammation [101]. Sham *et al.* synthesized *bis*-Schiff bases (**20**) made from guanidine and investigated them for anti-inflammatory activity; the results showed significant potential [102]. Also, Tantaru *et al.* formed *bis*-Schiff base (**21**) compounds resulting from acetophenone and established hopeful consequences when screening them for anti-inflammatory action [82].


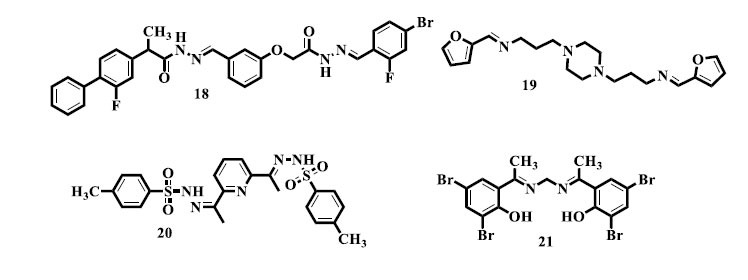


### 3.7 Urease Inhibitory Activity

The urease enzyme is responsible for catalyzing the alteration of urea into carbon dioxide and ammonia [103]. The alteration of urea, a leftover product of protein metabolism, into ammonia, which organisms can use as a source of nitrogen, plays a serious role in the nitrogen cycle [104]. Many organisms, such as fungi, bacteria, plants, and certain marine invertebrates, include urease [105]. Meanwhile, urease produces ammonia that can be injurious to cells in high absorption quantities and helps neutralize acidic situations; it is often associated with the capability of bacteria to colonize and infect crowded tissues. For example, the pathogenic bacterium *Helicobacter pylori* can cause gastric ulcers and further gastrointestinal ailments since it makes urease, which allows it to live and flourish in the stomach's acidic situation [106]. Urease has a role in nitrogen metabolism in plants, precisely in the reprocessing and remobilization of nitrogen. It is present in a diversity of plant tissues, such as the seeds, roots, and leaves. By altering urea into ammonia that may consequently be joined with other nitrogen-containing substances necessary for the growth and expansion of plants, it helps control the amount of nitrogen in the environment [107]. There is also a lot of interest in urease for its potential uses in agriculture and industry. Urease inhibitors are used in agriculture to control the hydrolysis of urea in soils, which lowers nitrogen losses due to ammonia volatilization and increases the efficiency of nitrogen utilization in fertilization techniques [108]. Urea is used in industry for procedures, like waste treatment, bioremediation, and ammonia-based product manufacturing.

Rashid *et al.* reported the synthesis of *bis*-Schiff base derivatives of ethyl phenyl ketone and screened them for their *in vitro* urease inhibitory potential, yielding a compound (**22**) as a promising anti-urease agent [109]. Novel isatin *bis*-Schiff base (**23**) derivatives were synthesized and reported by Pervez *et al.* as possible anti-urease drugs. The investigation of isatin-based compounds for their urease enzyme-inhibiting potential has benefited greatly from this work [110].


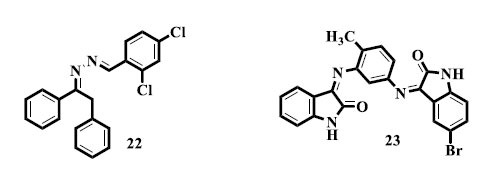


### 3.8 Cholinesterase Inhibitory Activities

Alzheimer's Disease (AD) is a neurological condition marked by behavioral and personality changes, memory loss, and a steady deterioration in cognitive function [111, 112]. It is the most typical cause of dementia in the senior citizen community. Although the precise origin of Alzheimer's disease is unknown, a mix of lifestyle, environmental, and genetic factors are thought to be involved [113, 114]. The buildup of aberrant protein aggregates, such as tau tangles and beta-amyloid plaques, in the brain is one of the main characteristics of Alzheimer's disease [115, 116]. These protein deposits impair neuronal communication and function, which causes the disease's hallmark symptoms. The breakdown of acetylcholine, a neurotransmitter important in memory, learning, and cognitive function, is largely dependent on cholinesterase enzymes [117, 118]. Acetylcholine is insufficient in Alzheimer's disease because cholinergic neurons in the brain are dying off. A class of drugs called cholinesterase inhibitors works by raising acetylcholine levels in the brain to treat Alzheimer's disease [119]. These medications decrease the breakdown of acetylcholine and enhance cognitive performance in some Alzheimer's patients by inhibiting the action of cholinesterase enzymes. Donepezil, rivastigmine, and galantamine are common cholinesterase inhibitors used in Alzheimer's disease treatment [120, 121]. These drugs do not change the fundamental course of the disease or stop its advancement, even though they can temporarily lower the rate at which cognitive decline develops in certain people and relieve their symptoms [122].

With encouraging outcomes, Ibrahim and associates reported the synthesis and cholinesterase inhibitory activity of new di-acetohydrazide *bis*-Schiff base (**24**) derivatives. According to the results, these compounds show promise in blocking cholinesterase enzymes, which may have consequences for the creation of medications that target cholinergic dysfunction in diseases, like Alzheimer's [123]. In addition, Taha *et al.* reported the synthesis and dual cholinesterase inhibitory actions of novel *bis*-Schiff bases (**25**) containing oxazoles, with encouraging outcomes [124]. Henia *et al.* synthesized novel symmetric azines (**26**) and successfully tested them for their *in vitro* cholinesterase inhibitory properties. This work has added to the body of knowledge regarding symmetric azines as putative cholinesterase inhibitors, so broadening the range of possible drugs for the treatment of conditions involving cholinergic dysfunction, including Alzheimer's disease [125]. Isatin *bis*-Schiff base (**27**) derivatives based on thiosemicarbazones were synthesized and tested for their ability to inhibit cholinesterase *in vitro*; they showed outstanding efficacy [126].


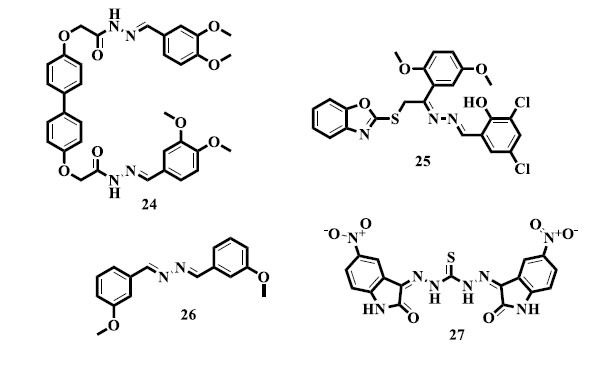


## CONCLUSION

We conclude that *bis*-Schiff-based compounds contain two azomethines (-CH=N-) functional groups and are effective in a number of fields, including agriculture, material science, organic synthesis, medicinal applications, and coordination chemistry. This review has highlighted the common synthetic methods and numerous biological and pharmacological activities connected with substituted azines/*bis*-Schiff bases. The potential of these molecules to act as enzyme inhibitors additionally improves their significance in medicinal and synthetic chemistry. Our systematic review may deliver a widespread understanding of azines/*bis*-Schiff bases, paving the way for upcoming research and invention in this field.

## AUTHORS’ CONTRIBUTIONS

S.M.N.A. and AF.A. contributed to the writing of the original draft and data collection; F.U.R. performed visualization; A.A.E and A.A. contributed to visualization and data collection. M.K. and M.A. performed writing, review, and editing.

## Figures and Tables

**Table 1:** Summary of synthetic methods for *bis*-Schiff bases along with their general parameters.

**Synthetic Methods**	**Catalyst**	**Yields (%)**	**Solvents**	**Time (hours)**	**Advantages**	**Disadvantages**
Acidic condition	CH_3_COOH, HCl, H_2_SO_4_	85-90	Mostly alcoholic	Reflux	Straightforward method and high yields	Potential side reactions due to strong acidic conditions
Ultrasonication condition	None	80-95	Mostly ethanol	2-4 mins	Environmentally friendly and quick process	Equipment dependency and optimization required
Microwave-assisted condition	Acetic acid	70-90	Any solvent	1-5 mins	Fast, efficient, and eco-friendly method and providing high yields	Limited control over the reaction conditions
Neutral condition	None	78-90	Any solvent	5-8	Simple and efficient	Longer reaction time and substrate scope

**Table 2:** Different azine derivatives along with the type of cancer.

**S. No.**	**Structure**	**Type**	**Mechanism of Action**
1	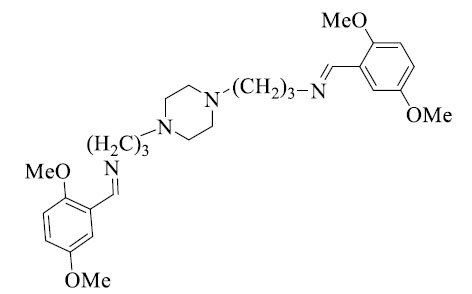	Lung cancer [52]	Induce apoptosis through caspase activation
2	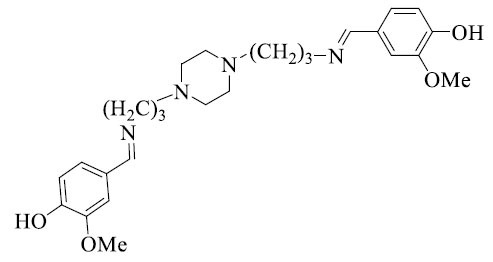	Breast cancer [52]	Stop cell proliferation
3	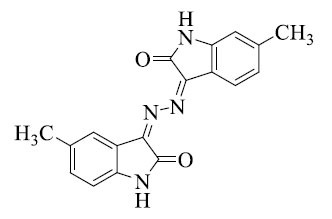	Cervical cancer [61]	Inactivate tumor suppressors Rb and p53 by HPV E6 and E7 proteins
4	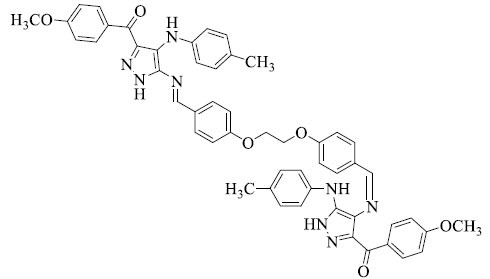	Breast cancer [62]	Stop cell proliferation
5	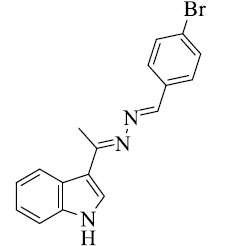	Colon cancer [63]	Modulate cell cycle
6	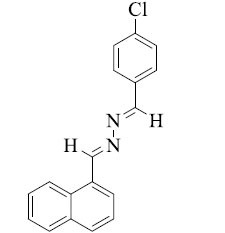	Liver cancer [64]	Inhibit specific kinases

## LIST OF ABBREVIATIONS

AGEs: Advanced Glycation End products
CDK: Cyclin-Dependent Kinases
RNS: Reactive Nitrogen Species
ROS: Reactive Oxygen Species
SAR: Structure-Activity Relationship
VEGF: Vascular Endothelial Growth Factor
